# A Lightweight Real-Time Vision Framework for Low-Disturbance Veterinary Management and Behavioral Welfare Assessment in Captive Forest Musk Deer

**DOI:** 10.3390/vetsci13101006

**Published:** 2026-09-23

**Authors:** Dequan Guo, Zijie Lan, Xin Fan, Xingyu Liu, Chuankang Chen, Chengli Zheng, Qiang Yang, Ferrante Neri

**Affiliations:** 1 School of Automation, Chengdu University of Information Technology, Chengdu 610225, China; guodq@cuit.edu.cn (D.G.);; 2 Sichuan Institute of Musk Deer Breeding, Sichuan Institute for Drug Control, Chengdu 611845, China; 3 Liberal Arts and Social Sciences, The Education University of Hong Kong (EdUHK), Hong Kong 999077, China

**Keywords:** captive forest musk deer, continuous behavior recording, non-contact monitoring, lightweight vision framework, conservation breeding, real-time detection

## Abstract

Non-contact behavioral monitoring is important for low-disturbance husbandry of captive forest musk deer, but variable illumination, shadows, partial occlusion, and low target-background contrast can reduce recognition reliability. This study developed HGS-YOLO26n, a lightweight real-time detector for continuous behavior recording under practical captive breeding conditions. HVIEnhanceStem was used to preserve shallow visual cues under weak illumination and low contrast, AdaGFB adapted feature extraction to posture-related shifts in local discriminative regions, and SDIoU improved bounding-box localization under occlusion and target-scale variation. The model was trained and evaluated using 6760 field images collected from 30 enclosures. HGS-YOLO26n achieved an mAP50–95 of 0.8409, exceeding the YOLO26n baseline by 3.74 percentage points, with 2.84 M parameters and an inference speed of 176.8 frames per second. Validation on continuous videos showed that its behavior-duration and event-count estimates were closer overall to blinded manual observations than those of the comparison models. By converting routine surveillance footage into longitudinal, individual-level records of behavior duration, event frequency, and temporal distribution, the system enables efficient review of behavioral activity across extended monitoring periods. Husbandry and veterinary staff can use these records to compare behavioral patterns across observation periods and return to corresponding video segments when specific changes require verification. This provides a low-disturbance source of quantitative behavioral information for routine husbandry, longitudinal observation, and conservation breeding of captive forest musk deer.

## Simple Summary

Forest musk deer are sensitive to human presence, but routine behavior monitoring still relies heavily on manual observation. This approach has two limitations: close observation may disturb the animals, and intermittent inspection is labor-intensive and cannot provide continuous records. Fixed-camera monitoring reduces direct human interference, but weak illumination, low contrast, partial occlusion, and posture changes can reduce recognition reliability. We therefore developed HGS-YOLO26n with three targeted improvements. HVIEnhanceStem strengthens visual cues under poor illumination and low contrast; AdaGFB adapts feature extraction to posture-related changes in local behavioral regions; and SDIoU stabilizes bounding-box localization under occlusion and target-scale variation. The model was evaluated using 6760 images from 30 forest musk deer enclosures and recognized four daily behaviors. It improved detection over the baseline while retaining lightweight real-time performance. In long videos, its behavior-duration and event-frequency records were also more consistent with blind manual observations. The system converts routine surveillance footage into structured behavioral records, reducing the need for full-length manual video review and providing longitudinal behavioral information for routine husbandry and veterinary observation.

## 1. Introduction

The forest musk deer is a protected wildlife species, and ex situ conservation breeding requires husbandry practices that minimize avoidable disturbance [1,2]. Longitudinal records of feeding, lying and resting, upright activity, and egestion characterize how individuals allocate their activity over time and provide quantitative information for routine behavioral observation [1,3]. Recent studies in captive musk deer have further reported associations between specific behavioral characteristics and fecal steroid hormones or reproductive outcomes [4,5], illustrating the broader biological relevance of behavioral monitoring in conservation breeding. Continuous, non-contact recording can therefore complement routine husbandry and veterinary observation by providing behavioral information across periods that are difficult to cover through direct observation alone.

In large forest musk deer breeding facilities, fixed cameras can continuously monitor many individually housed animals. However, the resulting video volume is difficult to review and record manually. The workload increases further when many enclosures must be monitored over days or longer periods. An automated system must therefore process multiple video streams efficiently over extended periods. Recognition must also remain reliable under weak illumination, shadows, low target-background contrast, partial occlusion, and posture variation. Wearable or contact-based sensors can provide continuous measurements, but their use requires additional handling and maintenance [6]. For facilities that already use fixed cameras, a software-based solution that reuses the existing surveillance infrastructure is more practical for large-scale, long-term monitoring.

Some vision-based methods obtain detailed behavioral information from spatial, anatomical, or temporal representations. SideCow-VSS preserves animal contours and spatial boundaries through semantic segmentation but requires pixel-level annotation [7]. Kim [8] estimated localized head and ear postures to capture finer anatomical cues. Our previous forest musk deer study used keypoint-based representations to distinguish subtle postural differences [9]. However, landmark annotation and pose inference increased the workload of model development and continuous deployment. Li et al. [10] combined RGB images, optical flow, and skeleton features to capture appearance and motion information. Nasiri et al. [11] used pose estimation with a CNN-LSTM network to model temporal posture changes. DeepLabCut provides flexible markerless pose estimation but still requires body-part annotation and an additional inference stage [12]. These methods provide rich spatial or temporal information. Their annotation and processing costs, however, make sustained parallel monitoring across many enclosure streams more demanding.

Lightweight frame-level methods reduce these costs and are more suitable for continuous deployment. Yao et al. [13] developed a compact model for real-time recognition of daily cattle behaviors. Méndez et al. [14] evaluated object detectors for direct localization and classification of goat behaviors. Xia et al. [15] applied YOLO to aggressive-behavior detection in piglets. These studies show that efficient frame-level monitoring is feasible. However, results from one species or farm setting may not transfer directly to forest musk deer enclosures. Illumination variation, occlusion, and limited cross-scene generalization remain common challenges in livestock vision systems [16]. IATEFF-YOLO addressed nighttime cow behavior detection under weak illumination [17]. DHSW-YOLO improved duck behavior recognition under bright and dark conditions [18]. Long-term monitoring also requires more than accurate predictions on individual frames. Missed detections can create gaps in continuous episodes. Class switches can alter accumulated duration, while unstable predictions can introduce false transitions or fragmented events. Temporal models such as CBLFormer can model behavioral transitions in video [19] but require consecutive-frame processing and additional temporal computation. Large-scale forest musk deer monitoring therefore requires robust frame-level recognition under complex enclosure conditions and reliable conversion of these predictions into behavior-duration and event-frequency records.

YOLO26n provides a compact end-to-end baseline for this task [20]. Low-light enhancement methods such as Zero-DCE show the value of improving visibility [21]. The HVI color representation also provides complementary intensity and chromatic information for low-light scenes [22]. Adaptive sampling has been used to improve feature extraction under non-rigid animal posture variation [23]. Deformable convolution provides the spatial flexibility needed to follow such local changes [24]. Geometry-aware IoU losses further improve bounding-box regression through overlap, position, and scale constraints [25,26,27]. HGS-YOLO26n was therefore designed with three corresponding components: HVIEnhanceStem for visibility adaptation, AdaGFB for posture-oriented feature extraction, and SDIoU for localization stability.

We integrated HGS-YOLO26n into a software-based behavior-recording framework for existing fixed-camera surveillance. The framework accepts directly connected cameras, RTSP streams, and stored videos without additional sensing hardware. It supports multi-source video processing and converts frame-level detections into records of behavior duration, event frequency, and temporal distribution. The system is intended to reduce full-length manual video review and support continuous monitoring across multiple enclosures.

Accordingly, this study addresses two research questions:

RQ1: Can HGS-YOLO26n improve four-class behavior detection under challenging enclosure conditions while retaining lightweight real-time inference?

RQ2: Can improvements in frame-level detection produce behavior-duration and event-frequency records that more closely agree with blinded manual references in long videos?

The study makes three methodological contributions. First, HVIEnhanceStem integrates detector-level RGB–HVI visibility adaptation into shallow feature extraction without requiring a stand-alone enhancement network. Second, AdaGFB couples learned offsets, local weighting, and dual residual fusion to adapt feature extraction to shifts in behavior-related anatomical regions. Third, SDIoU strengthens position- and scale-sensitive bounding-box regression before detections are converted into continuous records. We integrate the complete detector into our previously developed multi-source behavior-recording system and evaluate it at both the frame and long-video levels against blinded manual observations.

## 2. Materials and Methods

### 2.1. Animals, Enclosure Monitoring, and Dataset Construction

The dataset was retrospectively constructed from routine fixed-camera videos recorded at the Sichuan Institute of Musk Deer Breeding. It included 30 individually housed forest musk deer (15 males and 15 females) in 30 separate enclosures, each monitored by one fixed camera. The cameras were mounted at oblique overhead or top-down positions and operated under routine feeding, lighting, and husbandry schedules without additional handling, restraint, experimental stimulation, or environmental modification.

The video archive covered variations in animal posture, camera angle, enclosure background, camera-to-animal distance, illumination, and occlusion. Illumination conditions included daylight, dawn/dusk, artificial lighting, weak fill light, and nighttime infrared imaging, while common scene interference included corner shadows, fence occlusion, ground reflections, and low contrast between dark pelage and enclosure backgrounds.

Figure 1 summarizes the dataset-construction workflow. Candidate frames were selected to represent the four target behaviors and diverse illumination, posture, and occlusion conditions. Frames with severe blur, complete occlusion, indeterminate behavior, or excessive temporal redundancy were excluded. The retained images were resized to 640 × 640 pixels, resulting in a final dataset of 6760 images.

The task was formulated as single-frame object detection. Each image contained one forest musk deer and was annotated with a full-body bounding box and one mutually exclusive behavior label. The behavioral categories were developed with reference to an established forest musk deer ethogram [28] and the four-category framework used in our previous vision-based study [9], then refined for the present fixed-camera single-frame detection task. Table 1 summarizes the resulting operational definitions and criteria for handling borderline samples.

Bounding boxes covered the complete visible body, and annotation files were stored in YOLO format. Figure 2 shows representative fixed-enclosure views under different illumination conditions. To characterize illumination quantitatively, the mean luminance of the annotated deer region was calculated as Y = 0.299R + 0.587G + 0.114B and normalized to 0–1. For nighttime infrared images, the same normalized grayscale-based luminance measure was used. Table 2 reports the dataset split and class counts.

### 2.2. Dataset Partition and Class Distribution

After consensus review of all annotations, 5412 images were assigned to the training set and 1348 to the held-out validation set. To reduce similarity leakage, we grouped images jointly by behavior category and the annotated spatial position of the forest musk deer rather than randomly splitting individual frames. Each group was assigned exclusively to either the training or validation set. The validation set was used only for model evaluation, with no weight updates or hyperparameter adjustment.

Table 2 shows that Stand_or_walk and Lie_down_and_rest accounted for 42.07% and 27.38% of the retained labels, respectively, whereas egestion and Standing_feeding accounted for 15.50% and 15.04%. This distribution was broadly consistent with routine monitoring, in which upright activity and recumbent behavior occurred more frequently than egestion and feeding. The largest-to-smallest class ratio was approximately 2.80:1. We therefore retained the original class distribution without over- or under-sampling or class-dependent weighting in the classification loss.

### 2.3. Selection of the YOLO26n Baseline

YOLO26n was selected as the benchmark model to meet the operational requirements for long-term monitoring, real-time inference, and parallel processing of multiple video streams. Its end-to-end inference pipeline, lightweight regression head, and low parameter count provide a compact starting point for this use case [20]. The subsequent modifications focused on low illumination, low target-background contrast, and changes in local posture cues without substantially increasing computational demand.

### 2.4. The Architecture of HGS-YOLO26n

HGS-YOLO26n retains the backbone–neck–head organization of YOLO26n and introduces three targeted modifications (Figure 3). HVIEnhanceStem replaces the initial convolution layer at layer 0 for shallow visibility adaptation. AdaGFB replaces the corresponding backbone blocks at layers 2, 4, 6, and 8 for multi-scale posture-oriented feature extraction. The original end-to-end multi-scale detection head is retained, while SDIoU is applied to bounding-box regression for geometry-aware localization. The network outputs class probabilities and bounding boxes at each detection scale.

### 2.5. HVIEnhanceStem for Shallow Visibility Adaptation

The Horizontal/Vertical-Intensity (HVI) color space separates intensity from horizontal and vertical chromatic components for low-illumination representation [22]. HVIEnhanceStem incorporates this representation into shallow feature extraction through parallel RGB and HVI branches. The RGB branch preserves the original image information, whereas the HVI branch provides complementary intensity–chromaticity features for low-visibility regions. Each branch uses convolution, batch normalization [29], and nonlinear activation for shallow feature extraction.

Rather than applying HVI as independent image preprocessing, HVIEnhanceStem fuses RGB- and HVI-derived features within the detector. Given the wide illumination variation in the surveillance data, an adaptive gate controls their contribution to the fused representation. To distinguish the effect of detector-integrated adaptation from HVI transformation alone, we also constructed YOLO26n-HVIInput, in which the same HVI transformation defined in Equation (5) was applied to each input image before an otherwise unchanged YOLO26n.

For the RGB branch, the input image and its shallow features are defined as follows:

(1) $$X\in R^{H\times W\times 3}$$

(2) $$F_{rgb}=\phi_{rgb}\left(X\right)$$

For the HVI branch, the intensity map, horizontal and vertical chromatic components, and shallow HVI features are obtained using the HVI transformation as follows:

(3) $$I\left(x\right)=\underset{c\in \{R,G,B\}}{max}X_{c}\left(x\right)$$

(4) $$\hat{H_{c}}=C_{k}\left(I\right)\cdot S\cdot cos\left(\frac{\pi h}{3}\right),\;\hat{V_{c}}=C_{k}\left(I\right)\cdot S\cdot sin\left(\frac{\pi h}{3}\right)$$

(5) $$X_{hvi}=T_{hvi}\left(X\right)=Concat\left(\hat{H_{c}},\hat{V_{c}},I\right),\;F_{hvi}=\phi_{hvi}\left(X_{hvi}\right)$$

where X denotes the input RGB image; H and W are image height and width; c is the RGB channel index; *I*(*x*) denotes the intensity of pixel x; h and S denote hue and saturation; *C_k_*(*I*) is the intensity-dependent chromatic modulation coefficient; and *H_c_* and *V_c_* are the horizontal and vertical chromatic components in the HVI representation. In Equation (2), φ_rgb(·) denotes the shallow convolutional mapping of the RGB branch. In Equation (5), *T_hvi_* denotes the RGB-to-HVI transformation, *X_hvi_* is the HVI representation supplied to the HVI branch, $\phi_{hvi}$ (·) denotes its shallow convolutional mapping, and *F_rgb_* and *F_hvi_* are the features extracted from the two branches. The two feature tensors are then defined:

(6) $$F_{fuse}=\phi_{fuse}\left(Concat\left(F_{rgb},F_{hvi}\right)\right)$$

To avoid over-enhancement in normally illuminated regions, an adaptive gate is generated from the fused feature and applied to produce the final enhanced shallow feature:

(7) $$G=\sigma \left(\phi_{g}\left(F_{fuse}\right)\right)$$

(8) $$F_{out}=F_{fuse}⊙\left(1+G\right)$$

where Ffuse denotes the channel-fused feature, $\phi_{fuse}$ (·) denotes the fusion convolution, G is the gating weight, and $\phi_{g}$ (·) denotes the gating-branch mapping. The symbol σ is the sigmoid function, and ⊙ is element-wise multiplication. Fout is the enhanced shallow feature produced by HVIEnhanceStem. Its spatial dimensions and number of channels match the subsequent YOLO26n backbone, so the prediction logic of the neck and E2E detection head remains unchanged. Figure 4 shows the module structure. Figure 5 illustrates the intensity–chromaticity representation differences in representative low-illumination samples.

### 2.6. AdaGFB for Posture-Oriented Geometric Adaptation

Posture-dependent shifts in local behavior cues can reduce the reliability of fixed-grid feature sampling. Deformable convolution learns spatial offsets beyond fixed sampling grids [24], while attention-enhanced deformable sampling has shown benefits for non-rigid animal posture changes [23]. Building on these principles, we designed AdaGFB to combine offset prediction, deformable sampling, local feature weighting, and dual residual fusion in a lightweight block deployed at multiple backbone stages (Figure 6).

AdaGFB uses two residual additions after deformable sampling. The first combines the attention-weighted feature with the unweighted deformable feature to preserve useful responses, while the second restores the original input through an identity path. This design follows the general principle of residual identity mapping [30] while integrating it with adaptive sampling and local feature weighting.

Given an input feature X, AdaGFB first predicts sampling offsets and obtains the geometry-adaptive feature Z by deformable convolution. In parallel, a local-attention branch generates attention weights from X; these weights reweight Z before dual residual fusion. These operations are formulated as follows:

(9) $$\Delta p=\phi_{o}\left(X\right)$$

(10) $$Z=DConv\left(X,\Delta p\right)$$

(11) $$A=\sigma \;\left(\phi_{a}\left(X\right)\right)$$

(12) $$Z_{a}=A⊙Z$$

(13) $$Y=ReLU\left(Z_{a}+Z+X\right)$$

where $X\in R^{H\times W\times C_{1}}$ denotes the AdaGFB input feature; H and W are the spatial height and width of the feature map, and $C_{1}$ and $C_{2}$ denote the input and output channel dimensions, respectively. The offset-prediction mapping $\phi_{o}:R^{H\times W\times C_{1}}\to R^{H\times W\times 18}$ is implemented by a 3×3 convolution with stride 1 and padding 1; the 18 output channels encode the x - and y -direction offsets of nine sampling points in the 3×3 deformable kernel. $DConv\left(\cdot \right)$ denotes a 3×3 deformable convolution with stride 1 and padding 1, producing $Z\in R^{H\times W\times C_{2}}$. In parallel, the local-attention mapping $\phi_{a}:R^{H\times W\times C_{1}}\to R^{H\times W\times C_{2}}$ is implemented by a 1×1 convolution applied directly to X, followed by sigmoid activation to generate $A\in {\left(0,1\right)}^{H\times W\times C_{2}}$. The attention-weighted feature is $Z_{a}=A⊙Z$. In the AdaGFB instances used in this study, the residual path preserves the channel width ( $C_{1}=C_{2}=C$ ), consistent with Figure 6. Here, ⊙ denotes element-wise multiplication, $F_{1}=Z_{a}+Z$ denotes the first residual sum, and Y is the final output after addition of X and ReLU activation.

### 2.7. SDIoU Loss

Box localization affects both frame-level classification and cross-frame statistics. For egestion and Standing_feeding, the main cues lie near the hind limbs, hindquarters, and head-neck region. A displaced or poorly scaled box can weaken these cues and introduce gaps or inconsistencies in subsequent behavior records. To address this issue, SDIoU is used in the regression stage to dynamically balance overlap/aspect-ratio consistency and center-distance constraints according to target scale.

Here, $B_{p}=\left(x_{p},y_{p},w_{p},h_{p}\right)$ and $B_{g}=\left(x_{g},y_{g},w_{g},h_{g}\right)$ denote the predicted and ground-truth boxes, respectively, where $\left(x,y\right)$ represents the box-center coordinates and w and h denote box width and height. The IoU term, aspect-ratio term, target-scale coefficient, SDIoU score, and corresponding regression loss are defined as follows:

(14) $$IoU=\frac{\left|B_{p}\cap B_{g}\right|}{\left|B_{p}\cup B_{g}\right|+\varepsilon }$$

(15) $$v=\frac{4}{\pi^{2}}{\left[arctan\;\left(\frac{w_{g}}{h_{g}}\right)-arctan\;\left(\frac{w_{p}}{h_{p}}\right)\right]}^{2}$$

(16) $$\beta =min\;\left(\frac{w_{g}h_{g}\;\delta }{81},\;\delta \right),\;\;\delta =0.5$$

(17) $$SDIoU=\delta +\beta +\left(1-\delta +\beta \right)\left(IoU-\alpha v\right)-\left(1+\delta -\beta \right)\frac{\rho^{2}\left(b_{p},b_{g}\right)}{c^{2}}$$

(18) $$L_{SDIoU}=1-SDIoU$$

where $b_{p}=\left(x_{p},y_{p}\right)$ and $b_{g}=\left(x_{g},y_{g}\right)$ are the predicted- and ground-truth-box centers, respectively; $\rho^{2}\left(b_{p},b_{g}\right)$ is their squared Euclidean distance; and $c^{2}=c_{w}^{2}+c_{h}^{2}+\varepsilon$, where $c_{w}$ and $c_{h}$ are the width and height of the smallest enclosing box. The term v measures the aspect-ratio discrepancy, with $\alpha =v/\left(1-IoU+v+\varepsilon \right)$ as its adaptive coefficient; α is computed without gradient propagation in the implementation. The coefficient β is the target-scale coefficient. The constant 81 is the fixed area-normalization factor used in the implementation, δ=0.5 bounds the scale coefficient, and $\varepsilon =1\times 10^{-7}$ is used for numerical stability. The same δ and ε values were used in all experiments. $L_{SDIoU}$ was optimized jointly with the classification and other detection losses.

Compared with Complete Intersection over Union (CIoU), the implemented SDIoU introduces the target-scale coefficient β to adjust the relative contributions of overlap/aspect-ratio consistency and center-distance constraints. In HGS-YOLO26n, AdaGFB adapts feature sampling to posture-dependent regions, whereas SDIoU adjusts localization penalties according to target scale. The two components therefore act at different stages of localization under posture change, weak illumination, and partial occlusion.

### 2.8. Training Settings and Evaluation Metrics

All experiments were conducted on a 64-bit Windows 11 platform equipped with an AMD Ryzen 7 7435H processor (8 cores and 16 threads), 16 GB RAM, and an NVIDIA GeForce RTX 4050 Laptop GPU with 6 GB VRAM. The models were implemented in Python 3.10.20 using PyTorch 2.11.0, torchvision 0.26.0, CUDA 12.6, cuDNN 9.10.2, and Ultralytics 8.4.34, with NVIDIA driver version 581.29. All experiments used the same hardware and software environment. The main training and data-augmentation settings are summarized in Table 3. AMP denotes automatic mixed precision. Mosaic augmentation was disabled during the final 12 epochs so that final parameter refinement was performed on single-image samples that more closely matched the validation and inference distributions.

Detection performance and computational efficiency were evaluated using seven metrics: precision, recall, mAP50 (IoU threshold of 0.50), mAP50-95 (averages AP values over IoU thresholds from 0.50 to 0.95 in increments of 0.05), parameter count, GFLOPs, and FPS [31,32]. Precision, recall, IoU, and mAP were calculated with Equations (19)–(22):

(19) Precision = TP / (TP + FP)

(20) Recall=TP / (TP+FN)

(21) $$IoU=\left|B_{p}\cap B_{g}\right|/\left|B_{p}\cup B_{g}\right|$$

(22) $$mAP=\left(\frac{1}{C}\right)\sum AP_{i}$$

where TP, FP, and FN denote true positives, false positives, and false negatives, respectively; *AP_i_* denotes the area under the precision–recall curve for class i; and C is the number of classes. Compared with mAP50, mAP50-95 is more sensitive to localization errors and therefore provides a stricter assessment of box quality.

## 3. Results

### 3.1. Detection Results

Table 4 summarizes the frame-level detection performance of HGS-YOLO26n on the held-out within-facility validation set. The model achieved an overall precision of 0.9652, recall of 0.9322, mAP50 of 0.9721, and mAP50-95 of 0.8409. With 2.84 M parameters and approximately 5.86 GFLOPs for a 640 × 640 input, it reached 176.8 FPS under the adopted hardware and inference settings.

Class-level performance differed across the four behaviors. Lie_down_and_rest achieved the highest mAP50-95 (0.8928), whereas Stand_or_walk showed the lowest recall (0.8742) and mAP50-95 (0.7945). Standing_feeding had the lowest precision (0.9044). These differences are examined further through the class-specific error pathways in the normalized confusion matrix in Figure 7.

**Figure 7:**
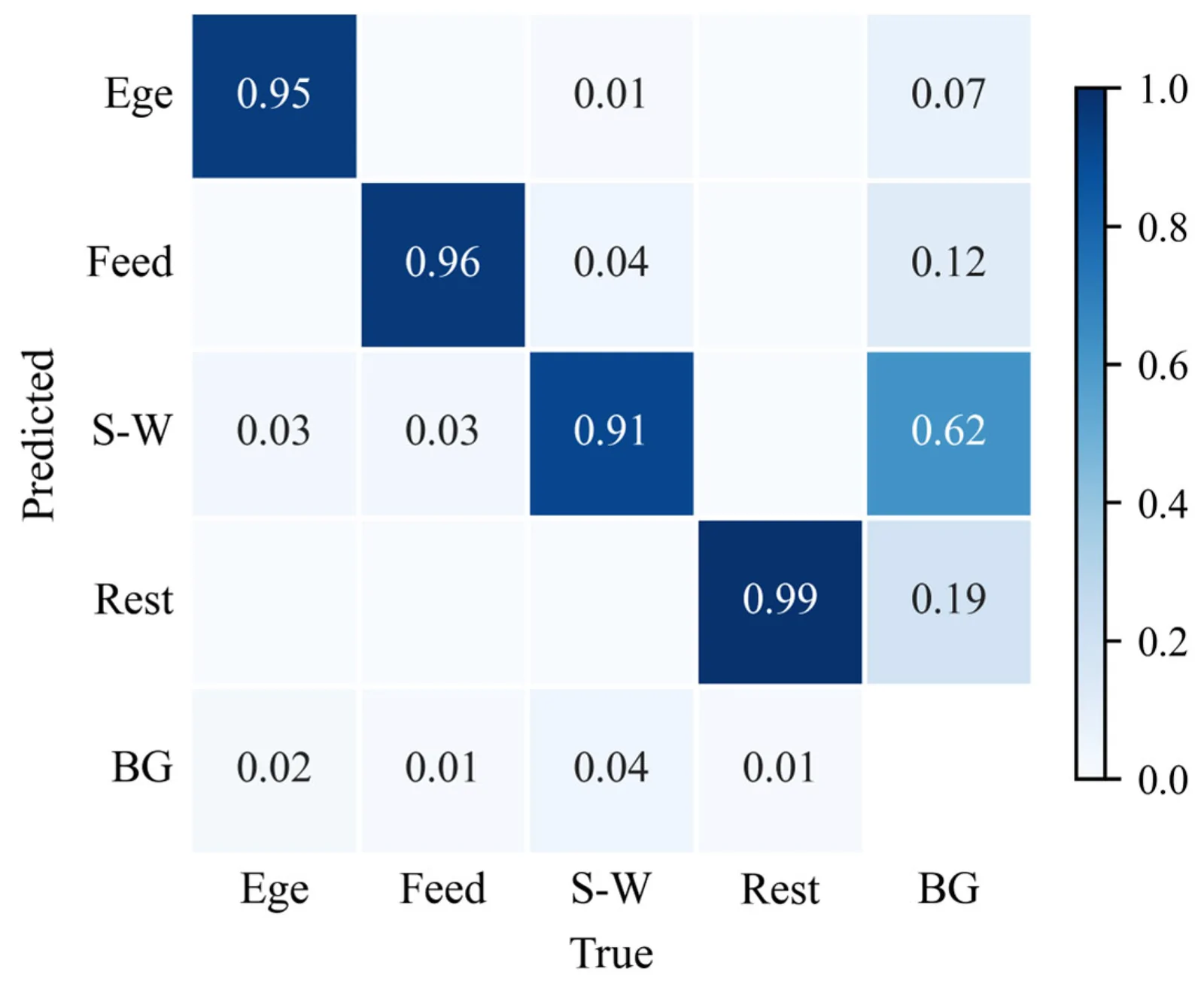
Normalized confusion matrix of HGS-YOLO26n.

Figure 8 shows the training and validation trajectories of box, classification, and DFL losses. All three losses decreased rapidly in the early epochs and then approached stable values, with only minor late-stage fluctuations in the validation curves. After Mosaic augmentation was disabled for the final 12 epochs, the training losses decreased more sharply, while the validation losses remained stable. This change is consistent with the transition from four-image Mosaic samples to single-image training inputs, which reduced training-sample complexity and made the final training inputs more consistent with those used during validation and inference. The resulting late-stage separation between the training and validation curves therefore reflects the change in augmentation regime rather than evidence of progressive overfitting. Overall, the loss curves indicate stable convergence.

Figure 7 presents the normalized confusion matrix and provides a class-specific analysis of the performance differences reported in Table 4. In the figure, Ege, Feed, S-W, Rest, and BG denote egestion, Standing_feeding, Stand_or_walk, Lie_down_and_rest, and background, respectively. The background row and column are not annotated behavior classes; they represent unmatched ground-truth instances and unmatched predictions, respectively.

Lie_down_and_rest showed the clearest separation, with 0.99 of its true instances correctly matched and classified. This result is consistent with its highest mAP50-95 (0.8928) in Table 4. Its distinctive low whole-body contour likely helps maintain class separability even when some local body regions are less visible.

The main inter-class ambiguity occurred between Standing_feeding and Stand_or_walk. Approximately 0.04 of true Stand_or_walk instances were assigned to Standing_feeding, whereas 0.03 of true Standing_feeding instances were assigned to Stand_or_walk. Both behaviors share an upright four-limb-supported body configuration and are distinguished mainly by local head-neck orientation and the muzzle–feeding-area relationship. Weakening of these local cues can therefore produce bidirectional confusion, consistent with the relatively low precision of Standing_feeding (0.9044) and lower recall of Stand_or_walk (0.8742) in Table 4.

Egestion showed a different error pattern, with 0.03 of its true instances assigned to Stand_or_walk. Its discriminative cues are concentrated around the hind limbs, hindquarters, and tail; weak illumination or partial occlusion may suppress these relatively small local cues while leaving the broader body posture visible. Unmatched ground-truth instances were also class dependent, accounting for approximately 2%, 1%, 4%, and 1% of true egestion, Standing_feeding, Stand_or_walk, and Lie_down_and_rest instances, respectively. Based on the validation-set class counts, these values correspond to an overall missed-detection proportion of approximately 2.4%. Among unmatched predictions, Stand_or_walk accounted for the largest share (62%), followed by Lie_down_and_rest (19%), Standing_feeding (12%), and egestion (7%). This distribution suggests that uncertain foreground-like regions were more likely to be assigned to the broader upright-activity category.

### 3.2. Ablation Experiments

To isolate the contribution of each component, YOLO26n was used as the baseline, and five controlled variants were evaluated under identical training and evaluation settings, as shown in Table 5. YOLO26n-HVIInput applied HVI transformation as independent preprocessing, whereas YOLO26n-HVIEnhanceStem integrated RGB-HVI adaptation into the detector. YOLO26n-SDIoU retained the original architecture and replaced only CIoU with SDIoU. YOLO26n-AdaGFB + SDIoU added adaptive geometric feature extraction, and HGS-YOLO26n combined complete visibility and geometry pathways. The variants were arranged progressively so that adjacent configurations differed by one modification, enabling the marginal contribution of each component to be assessed.

**Table 5:** Ablation results for the proposed components and HVI preprocessing.

Model	Loss	Params	P	R	mAP50	mAP50-95	FPS
YOLO26n	CIoU	2,375,616	0.9432	0.9084	0.9528	0.8035	214.3
YOLO26n-HVIInput	CIoU	2,375,616	0.9451	0.9135	0.9565	0.8105	190.2
YOLO26n-HVIEnhanceStem	CIoU	2,376,264	0.9518	0.9256	0.9654	0.8276	211.8
YOLO26n-SDIoU	SDIoU	2,375,616	0.9421	0.9156	0.9562	0.8108	214.3
YOLO26n-AdaGFB + SDIoU	SDIoU	2,843,604	0.9395	0.9248	0.9607	0.8162	182.7
HGS-YOLO26n	SDIoU	2,844,252	0.9652	0.9322	0.9721	0.8409	176.8

*Note: FPS for YOLO26n-HVIInput includes both independent HVI transformation and YOLO26n inference.*

Independent HVI preprocessing produced a modest improvement over the baseline, whereas the detector-integrated YOLO26n-HVIEnhanceStem achieved a higher mAP50-95 of 0.8276 and retained 211.8 FPS, compared with 0.8105 and 190.2 FPS for YOLO26n-HVIInput. HVIEnhanceStem added only 648 parameters, approximately 0.03% of the baseline model. These results indicate that the improvement was not attributable to HVI transformation alone; integrating complementary RGB-HVI features within the detector provided more effective visibility adaptation while avoiding an additional serial preprocessing stage.

Replacing CIoU with SDIoU increased recall from 0.9084 to 0.9156 and mAP50-95 from 0.8035 to 0.8108, while precision remained nearly unchanged. The improvement was greater for the stricter mAP50-95 metric than for mAP50, consistent with the geometry-sensitive constraints introduced by SDIoU. Adding AdaGFB further increased recall to 0.9248 and mAP50-95 to 0.8162, accompanied by a slight decrease in precision. This pattern suggests improved recovery of posture-variable instances through adaptive geometric sampling, with a limited precision-recall trade-off.

HGS-YOLO26n achieved the best overall performance, with a precision of 0.9652, recall of 0.9322, and mAP50-95 of 0.8409. Adding HVIEnhanceStem to the AdaGFB + SDIoU pathway improved both precision and recall rather than extending the recall gain alone, supporting the complementary effects of the visibility and geometry pathways.

### 3.3. Comparison with Real-Time Detectors

To compare HGS-YOLO26n with representative real-time detectors, this study evaluated SSDLite-MobileNetV2, EfficientDet-D0, YOLOX-nano, YOLOX-tiny, PP-YOLOE-s, RTMDet-tiny, RT-DETR-R18, YOLOv5n, YOLOv8n, YOLO11n, and the baseline YOLO26n. The comparison covers ultra-lightweight CNN detectors [33,34,35], lightweight YOLO-series detectors [20,36,37,38,39], and a real-time transformer detector [40], encompassing different accuracy, model-complexity, and inference-speed profiles.

All comparison models used the same dataset split, input size, training settings, and evaluation procedure. Table 6 reports their validation results. Params denotes parameter count, GFLOPs denotes computation for a 640 × 640 input, and FPS denotes inference speed.

The comparison shows a clear accuracy–efficiency trade-off. Speed-oriented detectors, particularly YOLOX-nano, achieved higher inference speed but substantially lower mAP50-95. In contrast, RT-DETR-R18 required 20.01 M parameters and 59.96 GFLOPs without exceeding the mAP50-95 of HGS-YOLO26n. Relative to YOLO26n, HGS-YOLO26n increased the parameter count from 2.38 M to 2.84 M and reduced FPS from 214.3 to 176.8, while mAP50-95 increased by 3.74 percentage points. The proposed model therefore retained real-time inference while providing a favorable accuracy–complexity balance for the intended multi-camera monitoring front end. Figure 9 illustrates representative outputs under weak illumination, shadows, and partial occlusion. In the selected examples, HGS-YOLO26n retained more complete target coverage and correct behavior labels, whereas the comparison models showed box displacement, incomplete coverage, or class confusion.

### 3.4. Application-Level Validation of Continuous Behavior Recording

For application-level validation, HGS-YOLO26n was integrated into the forest musk deer behavior detection and recording system previously developed by our team to process surveillance streams from captive enclosures. The validation examined whether improvements in frame-level detection translated into duration and event-frequency records that more closely matched blinded manual reference annotations.

As shown in Figure 10, the system follows a configuration–execution–analysis workflow. After loading the model weights and selecting a directly connected camera, RTSP stream, or local video file, it can operate in real-time single-channel, multi-source parallel, or offline batch-processing mode. End-to-end latency and concurrent-stream capacity depend on the loaded model, input-video configuration, hardware resources, and decoding and visualization overhead; therefore, no fixed maximum channel capacity is assigned to the software framework. The system performs frame-level object and behavior detection, caches the detection results, and converts the resulting sequence into behavioral event segments, Gantt charts, frequency statistics, time proportions, and structured data tables according to predefined confidence thresholds and duration-filtering rules.

Figure 11 presents multi-camera interface for monitoring multiple enclosures. Inference outcomes from the model are visually marked on surveillance videos via bounding rectangles, behavioral tags, and corresponding confidence level, while the control panel displays the current ongoing behavior, its accumulated duration, and the time proportion of each recorded behavior category. Figure 12 presents the behavioral timelines, event frequencies, and time proportions generated by the recording system. These outputs summarize continuous surveillance videos into structured behavior records, allowing users to review behavioral changes across different enclosures, observation periods, and behavior categories.

Musk deer may be temporarily occluded, hidden by enclosure structures, or outside the usable camera view, so the total video duration can exceed the interval during which behavior is reliably assignable. In model-generated records, low-confidence or missing detections were left unassigned rather than forcibly classified. For the manual reference, intervals with severe occlusion, absence from the effective field of view, severe blur, or ambiguous transition postures were marked as undetermined and excluded from behavior-duration assignment.

To evaluate whether frame-level improvements translated into system-level recording quality, five long videos from different enclosure surveillance records were analyzed using the multi-source mode of the behavior detection and recording system. V1 was recorded in enclosure 1-1-1 from 12:19 to 14:34 on 7 July, 2025; V2 in enclosure 1-3-2 from 07:58 to 10:38 on 8 July, 2025; V3 in enclosure 1-5-5 from 15:05 to 17:34 on 9 July, 2025; V4 in enclosure 1-4-1 from 11:11 to 13:21 on 10 July, 2025; and V5 in enclosure 2-1-6 from 23:55 on 7 July to 02:55 on 8 July, 2025. Four videos represented conventional surveillance conditions, whereas V5 was recorded under nighttime weak-light infrared imaging. As shown in Table 7, each video lasted 2–3 h.

Manual reference records were established through a structured multi-reviewer procedure. The reviewers were experienced forest musk deer keepers and breeding specialists familiar with the animals’ daily behavioral characteristics. Two annotators independently reviewed the five long videos and recorded behavior duration and event count, and all reviewers were blinded to the model-generated detections, timelines, and summary statistics. When the relative difference in the estimated duration of a behavioral segment between the two annotators was no greater than 5%, the third reviewer examined the corresponding video segment and determined the final reference record. When the difference exceeded 5%, all three reviewers jointly re-examined the relevant consecutive frames and local posture changes and reached a consensus before the record was finalized. This procedure was used to reduce individual judgment bias and improve the consistency of the manual reference.

The duration records shown in Figure 13 were obtained after confidence-threshold filtering, temporal merging, and duration calculation. The processed outputs were summarized at 30 s intervals for comparison with the blinded manual references, and event counts were defined as continuous behavior episodes after temporal merging. Duration and event count were evaluated separately to preserve their direct interpretation at the application level.

Figure 13 shows that HGS-YOLO26n produced behavior records closest to the blinded manual reference across five long videos. Its four recorded durations are summarized to 683.5 min, compared with 686.0 min for the manual reference and 679.0 min for YOLO26n. However, total recorded time alone does not fully characterize recording quality because overestimation of one behavior can offset underestimation of another. HGS-YOLO26n also reduced class-specific duration deviations across all four behaviors, indicating more accurate allocation of recorded time rather than merely more complete detection.

Lie_down_and_rest showed relatively small deviations across the compared models. Its long episodes, stable low-body contour, and limited number of transitions provide persistent whole-body cues across consecutive frames. Consequently, isolated missed or incorrect predictions affect only a small proportion of accumulated duration and can often be absorbed by temporal merging without creating additional event boundaries. This helps explain the comparatively stable duration and event-count estimates for this behavior.

A different pattern was observed for the three upright or locally defined behaviors. Most comparison models overestimated Stand_or_walk while underestimating Standing_feeding and egestion. As indicated in Figure 7, Standing_feeding depends on local head-neck and muzzle–feeding-area cues, whereas egestion relies more on hindquarter cues; when these cues are weakened by illumination, occlusion, or posture variation, the broader upright posture may remain visible and predictions can shift toward Stand_or_walk. HGS-YOLO26n reduced the excess Stand_or_walk duration while recovering more of the missing Standing_feeding and egestion duration, indicating more accurate allocation of behavior time among these visually related classes.

Missed and false detections provide a second pathway by which frame-level errors propagate into long-video records. Figure 7 showed an overall missed-detection proportion of approximately 2.4% on the validation set, while Stand_or_walk accounted for the largest share (62%) of unmatched predictions. A missed detection can create a gap within a continuous episode and, if the gap exceeds the temporal-merging tolerance, shorten the accumulated duration or split one event into multiple segments. Conversely, an unmatched prediction can introduce a spurious interval or additional event. Figure 13 similarly shows larger event-count deviations for shorter or more transitional behaviors, whereas Lie_down_and_rest remained comparatively stable because its long episodes were less sensitive to isolated frame-level errors. Together, these results indicate that the frame-level improvements reduced both behavior-time misallocation and recording errors caused by missed, false, and unstable consecutive predictions.

## 4. Discussion

### 4.1. Principal Findings

The principal finding of this study is that improved continuous behavior recording resulted from the complementary effects of visibility adaptation, posture-oriented feature extraction, and geometry-aware localization rather than from image-level accuracy alone. The ablation results showed that HVIEnhanceStem improved access to discriminative cues under poor visibility, whereas AdaGFB and SDIoU improved the recovery and localization of posture-variable instances. These complementary effects were also reflected in the long-video evaluation, where HGS-YOLO26n produced duration and event-count records closer to the blinded manual reference.

The behavior-dependent results further illustrate how frame-level errors propagate into longitudinal records. Lie_down_and_rest remained comparatively stable because its persistent whole-body contour provided robust cues across consecutive frames, whereas Standing_feeding and egestion relied more on smaller local cues and were therefore more susceptible to reassignment toward Stand_or_walk or to missed detections. Consecutive misses can create gaps or split continuous events, while class switching reallocates recorded duration among behavior categories. These findings indicate that improving visibility, adaptive feature extraction, and localization stability at the detector level can reduce the propagation of frame-level errors into continuous behavior records.

Previous studies show different trade-offs between behavioral detail and deployment efficiency. Yao et al. [13] developed a lightweight cattle behavior recognition model for standing, lying, and eating, showing that daily behavior recognition can be implemented with a compact real-time network. Méndez et al. [14] evaluated CNN- and Transformer-based detectors for eating, standing, drinking, and lying in goats under both light and dark conditions. Their multi-object framework is well suited to group-housed animals and provides direct behavior localization. However, the evaluation was conducted within a single farm and fixed top-view configuration and did not extend frame-level detections to temporal behavior records. HGS-YOLO26n instead targets individually housed forest musk deer under fixed-camera surveillance, with emphasis on lightweight inference and recognition under weak illumination, occlusion, and posture variation.

Video-based methods provide another direction. Hu et al. [19] proposed CBLFormer to recognize chicken behaviors around behavioral transitions and localize their temporal boundaries using spatiotemporal features. Such temporal modeling provides richer information on behavior transitions but requires consecutive video segments and additional temporal processing. HGS-YOLO26n instead serves as a lightweight visual front end for long-term surveillance across multiple enclosure streams. With 2.84 M parameters and 176.8 FPS on the adopted hardware, it retains the computational efficiency required for multi-stream monitoring. The present study also extends evaluation beyond frame-level metrics by examining whether detection improvements are retained after predictions are converted into behavior-duration and event-count records in long videos.

### 4.2. Management Implications for Captive Forest Musk Deer

The system converts routine multi-enclosure surveillance videos into structured records of behavior duration, event count, time proportion, and temporal distribution. Breeding and veterinary staff can use these summaries to identify periods that require closer review and return directly to the corresponding video clips. This reduces the need to inspect full-length recordings. The framework is entirely software-based and can reuse existing fixed-camera surveillance, RTSP streams, and stored videos without additional sensing hardware. It therefore supports long-term monitoring without wearable sensors, restraint, or extra handling solely for behavioral observation.

Behavioral information is relevant to conservation breeding because captive management involves long-term observation of animal adaptation, husbandry conditions, and individual welfare. Lu et al. [41] reported associations between stereotypic behavior, personality-related traits, adaptation to captivity, and musk-related characteristics in captive forest musk deer. Zhou et al. [42] examined social stress together with stereotypic behavior, fecal cortisol, and musk secretion in captive Alpine musk deer. These studies illustrate the broader biological relevance of longitudinal behavior records. They also show that behavioral observations should be interpreted together with physiological, environmental, and husbandry information.

The four categories recorded in this study describe complementary components of daily activity allocation. Standing_feeding records time spent in an identifiable feeding posture, so sustained changes in its duration or frequency can prompt review of feeding provision, feeding schedules, or the corresponding video. Lie_down_and_rest describes recumbent and resting allocation and can be compared across observation periods. Stand_or_walk summarizes upright activity, whereas egestion records elimination-related episodes. When accumulated longitudinally, changes in the duration, frequency, or temporal distribution of these behaviors can help staff select periods for closer inspection and video verification. Because similar behavioral changes may arise under different conditions, their interpretation is most meaningful when considered together with individual history, enclosure conditions, feeding records, and veterinary observations.

### 4.3. Limitations and Future Directions

A key limitation is that the dataset was collected at a single breeding facility with a limited range of fixed-camera configurations. Field visits and project collaborations in Sichuan, Shaanxi, and Xizang suggest that large forest musk deer facilities often use similar individual-enclosure layouts and fixed-camera surveillance. Even so, the present results reflect within-facility performance and do not demonstrate cross-site generalization. Future work will test the current model directly on data from independent breeding facilities with different enclosure layouts, camera viewpoints, seasons, and illumination conditions. If performance decreases at a new site, lightweight domain adaptation or site-specific fine-tuning will be evaluated using a limited amount of labeled target-site data. The adapted model will then be tested on held-out data from that site. Continual learning may also be explored as monitoring conditions change over time.

A further limitation is that Stand_or_walk combines stationary standing, slow walking, and brief upright transitions. This makes single-frame annotation more reliable, but it may hide temporal patterns such as repetitive pacing or restlessness. Future work could use adjacent frames, movement trajectories, speed, and short-term behavioral states to separate standing from walking and identify repetitive locomotor patterns. Temporal information may also reduce gaps in behavioral records during brief occlusion or posture changes. Behavioral records could then be combined with feeding logs, environmental data, veterinary observations, physiological and endocrine measurements, musk secretion, and reproductive outcomes. These data would help determine the biological and management significance of sustained behavioral changes.

With the accumulation of such longitudinal multimodal data, a further direction is to develop intelligent assessment methods that jointly interpret behavioral, environmental, physiological, and veterinary information. The behavioral records could be linked with our previously developed forest musk deer veterinary knowledge graph [3], providing structured veterinary knowledge for higher-level interpretation. On this basis, multimodal foundation models, including vision-language models, could be explored as reasoning tools for integrating surveillance images, behavioral timelines, and structured veterinary information to support the contextual interpretation of longitudinal behavioral changes.

## 5. Conclusions

This study developed HGS-YOLO26n for continuous, non-contact behavioral monitoring of captive forest musk deer. The proposed visibility adaptation, posture-oriented feature extraction, and geometry-aware localization improved frame-level behavior recognition while retaining lightweight real-time inference. These improvements were also reflected in continuous video analysis, where the resulting behavior-duration and event-count records showed closer agreement with blind manual observations. Integration with the multi-source monitoring system further enabled routine surveillance footage to be converted into structured longitudinal behavioral records. By linking frame-level computer-vision performance with the reliability of downstream behavioral records, this study provides a practical approach for efficient, low-disturbance monitoring of captive forest musk deer. The resulting records can complement routine husbandry and veterinary observations by providing quantitative descriptions of behavioral activity across extended monitoring periods, with broader potential for conservation breeding and smart animal-monitoring systems.

## Figures and Tables

**Figure 1:**
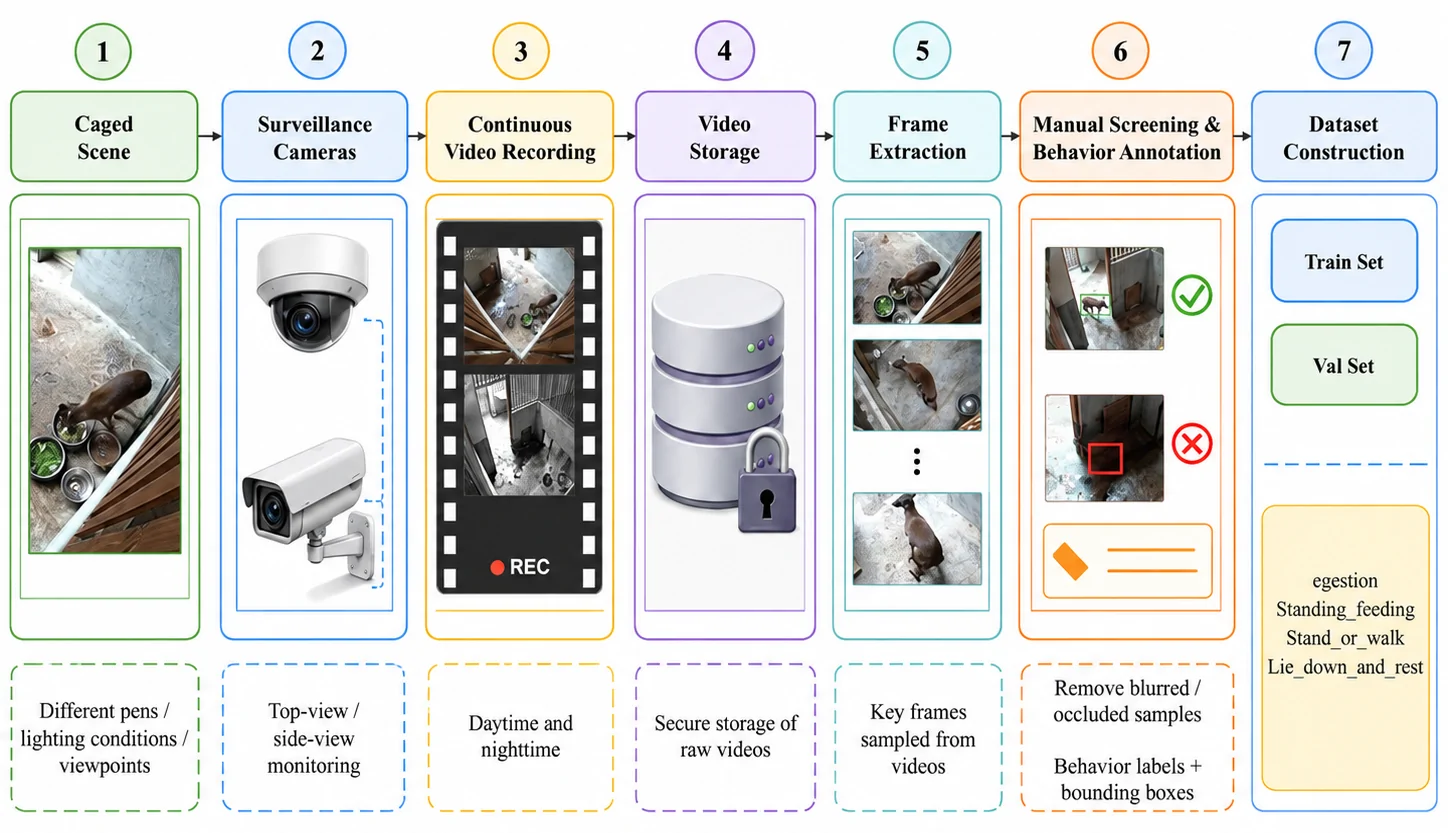
Surveillance video collection and dataset construction.

**Figure 2:**
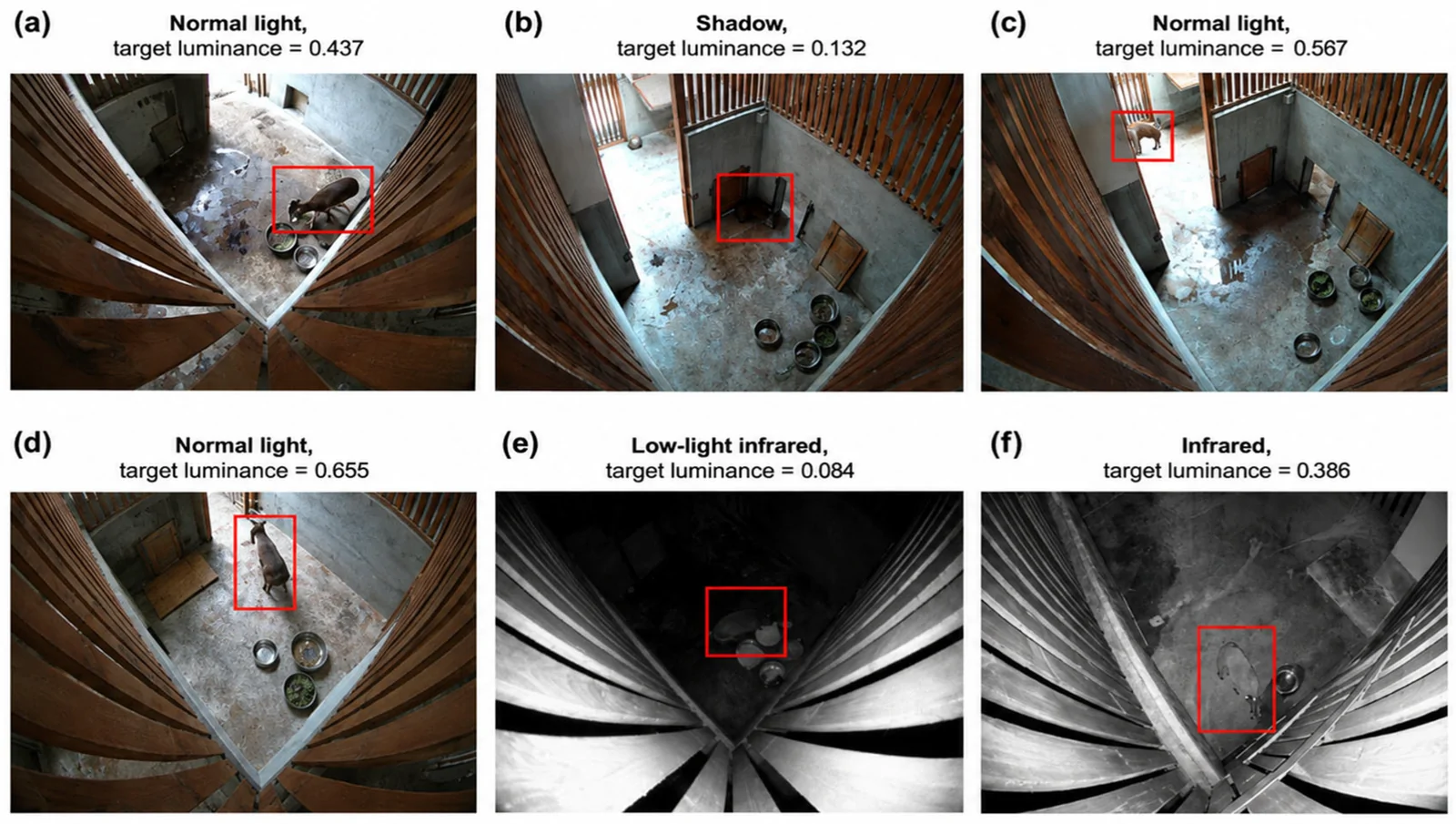
Representative fixed-enclosure surveillance views under different illumination conditions. (**a**) Normal-light scene, target luminance = 0.437; (**b**) shadowed scene, target luminance = 0.132; (**c**) normal-light scene, target luminance = 0.567; (**d**) normal-light scene, target luminance = 0.655; (**e**) low-light infrared scene, target luminance = 0.084; and (**f**) infrared scene, target luminance = 0.386. Red boxes indicate the approximate locations of the forest musk deer for visual reference only and do not represent model predictions.

**Figure 3:**
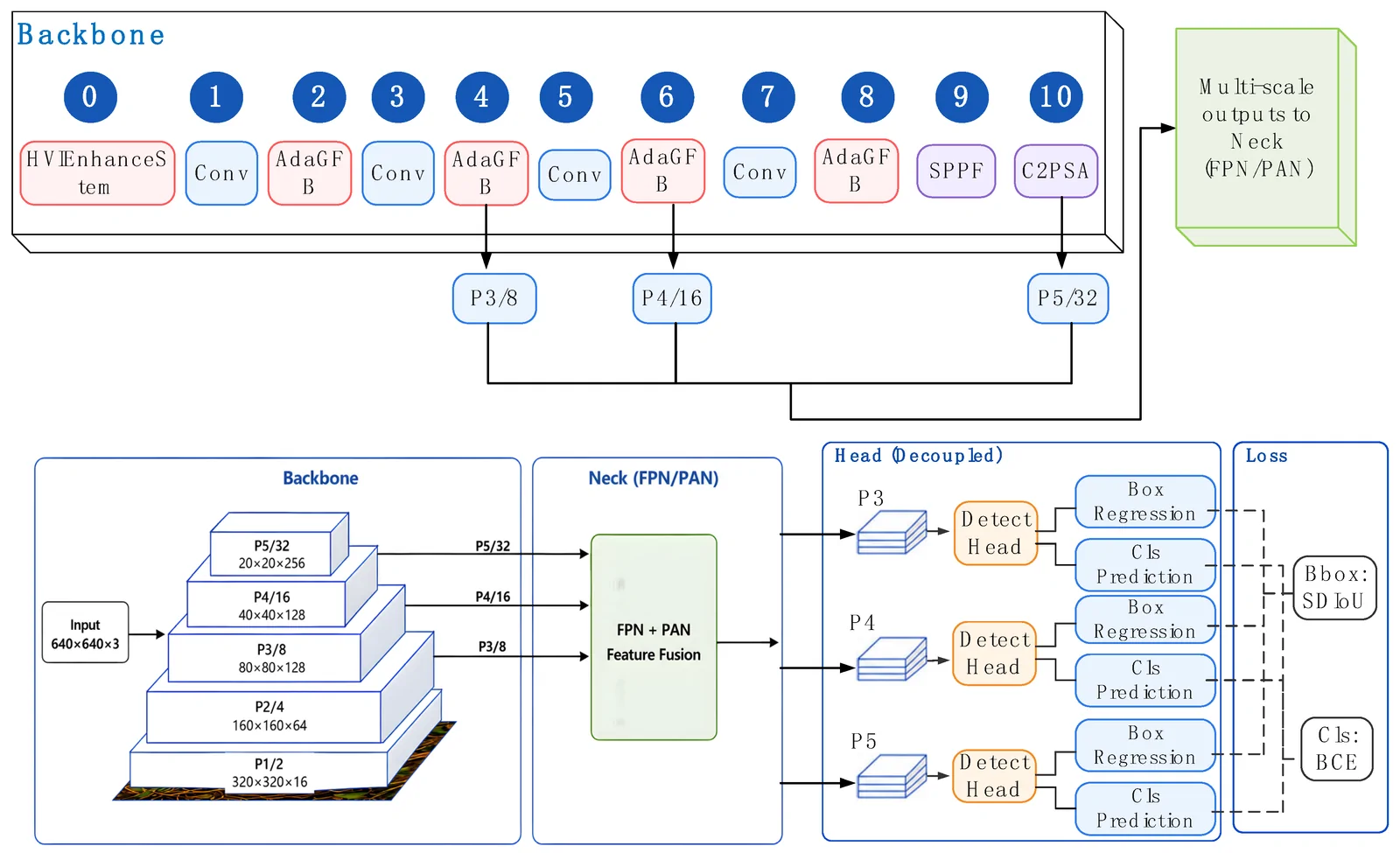
Overall architecture of HGS-YOLO26n.

**Figure 4:**
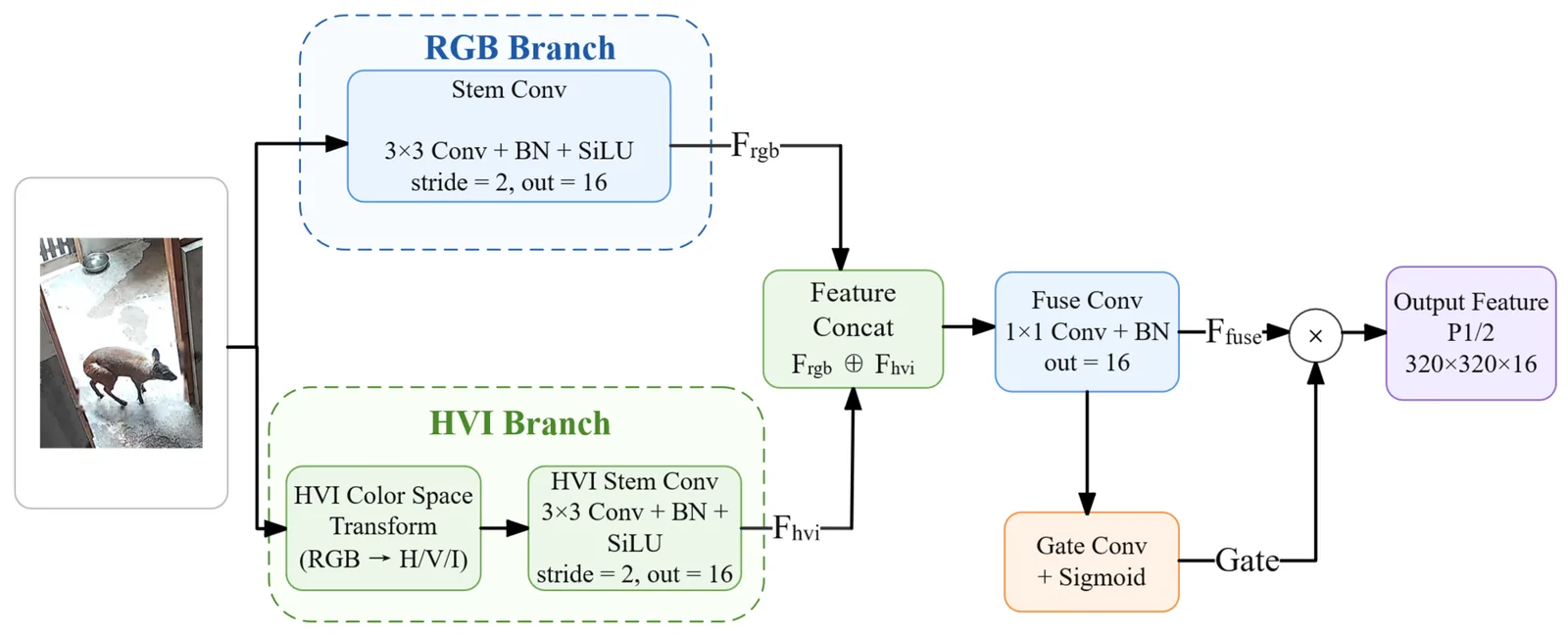
Structure of the HVIEnhanceStem module. ⊕ denotes feature concatenation, and × denotes element-wise multiplication.

**Figure 5:**
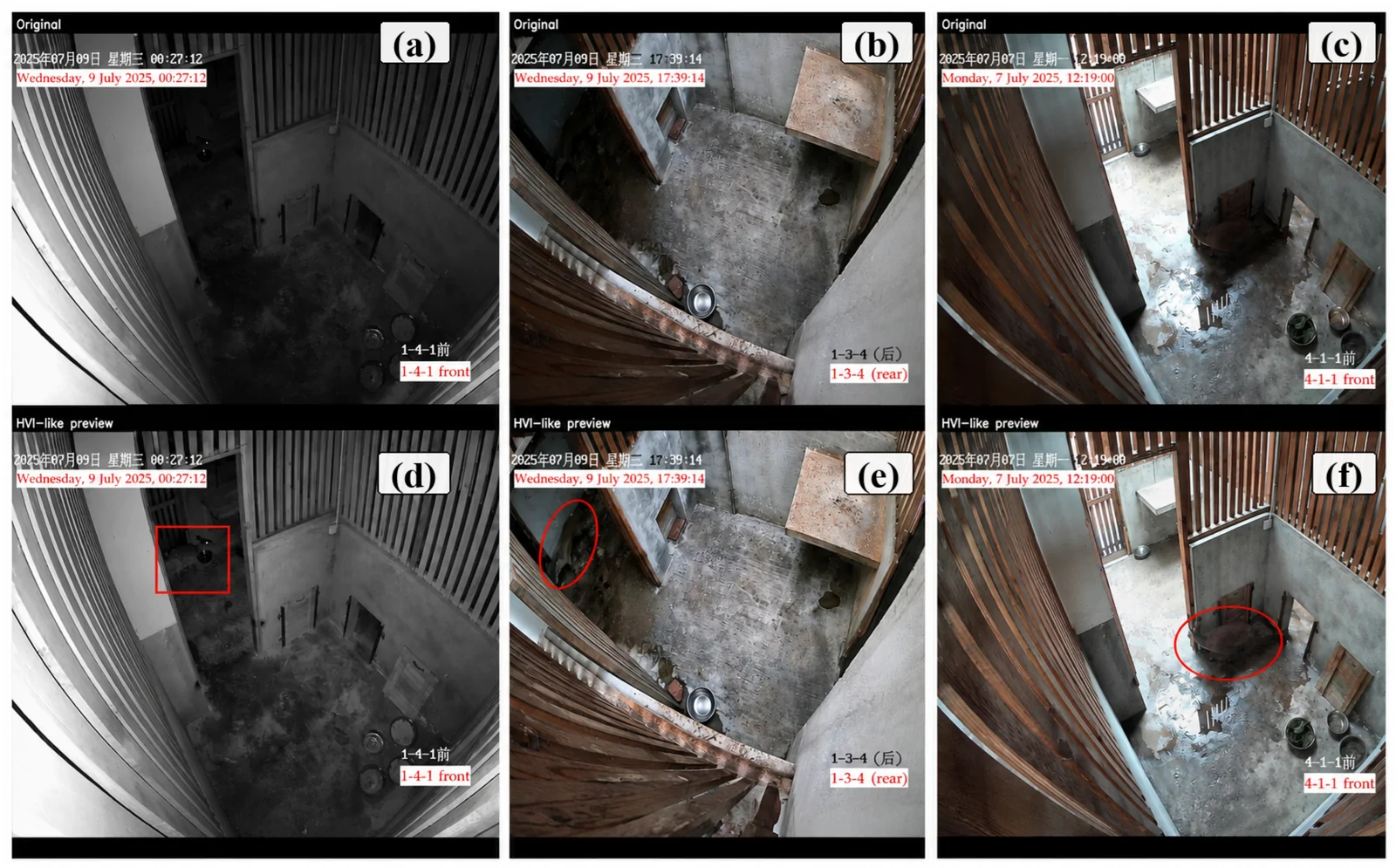
Qualitative visualization of HVI-based visibility changes in representative low-illumination surveillance samples. (**a**) Original low-light infrared image of Sample 1; (**b**) original low-illumination image of Sample 2; (**c**) original image of Sample 3 with the forest musk deer located in a shadowed region; (**d**) HVI-like visibility preview corresponding to (**a**); (**e**) HVI-like visibility preview corresponding to (**b**); and (**f**) HVI-like visibility preview corresponding to (**c**). The red box and circles indicate the approximate locations of the forest musk deer for visual reference only and do not represent model predictions.

**Figure 6:**
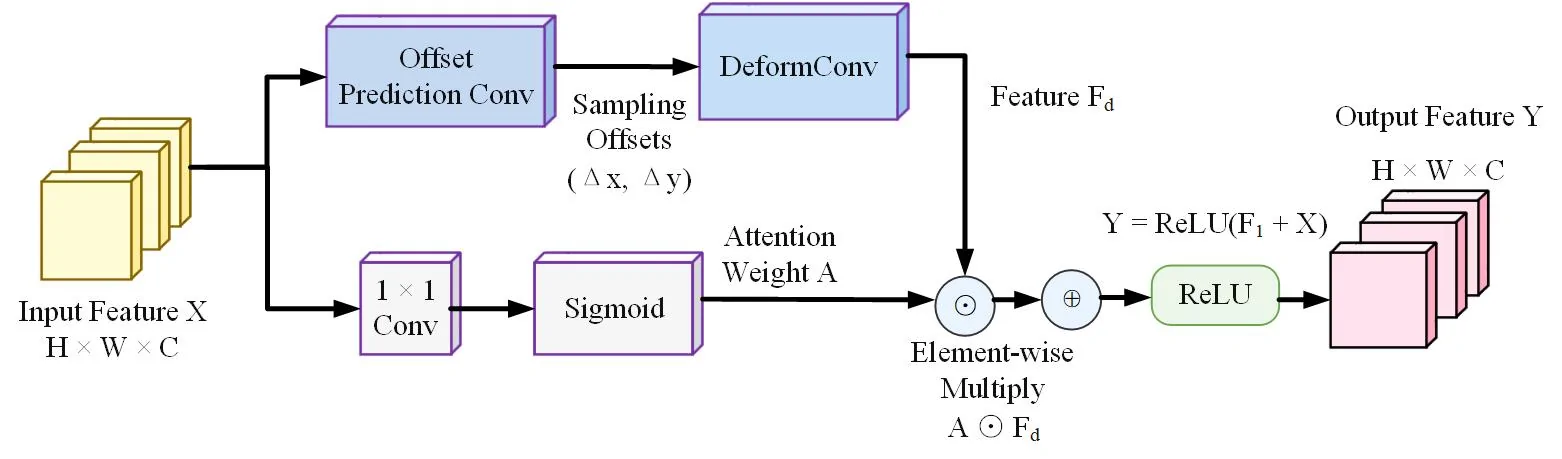
Adaptive Geometric Feature Block (AdaGFB).

**Figure 8:**
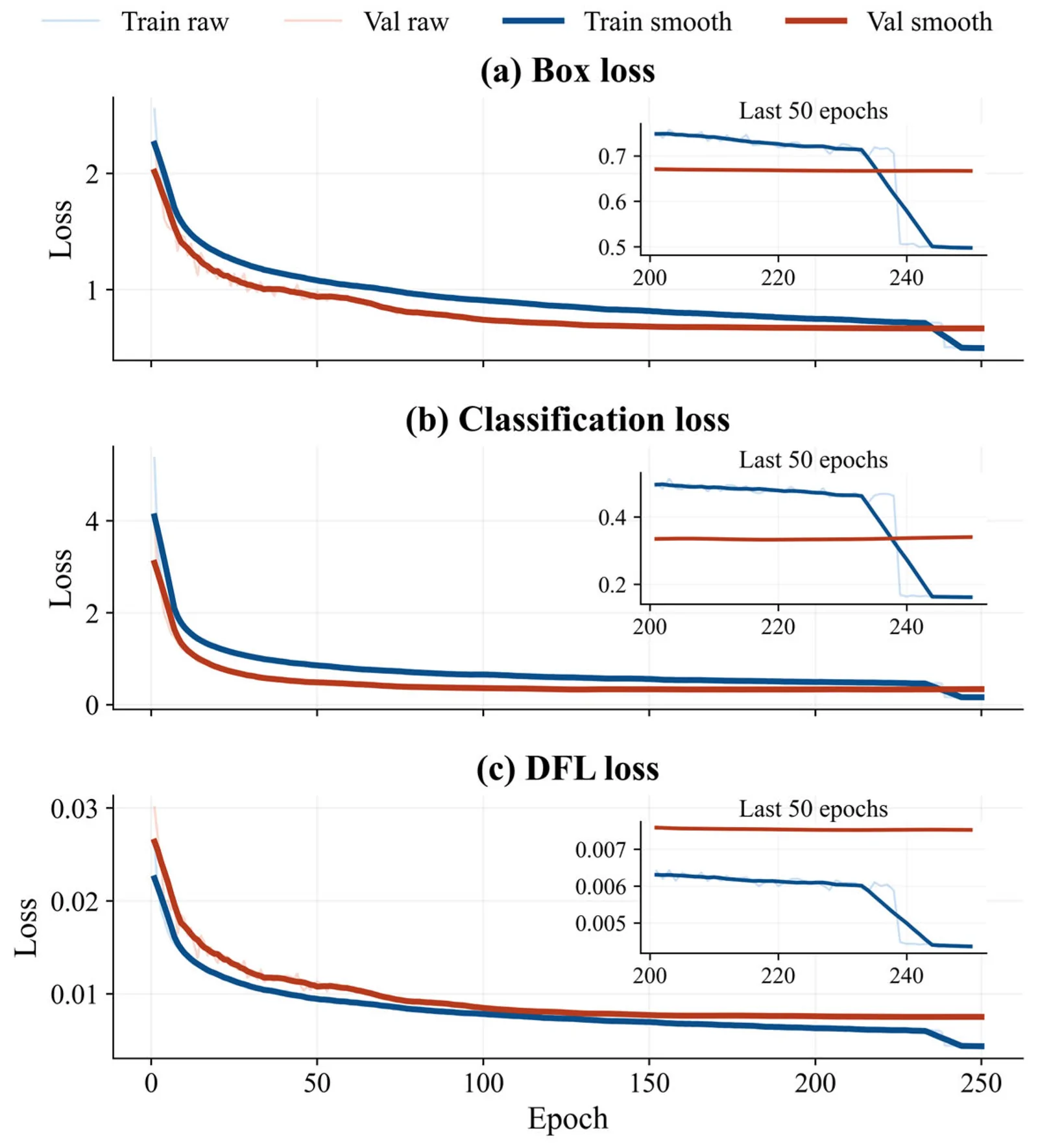
Training and validation loss curves of HGS-YOLO26n.

**Figure 9:**
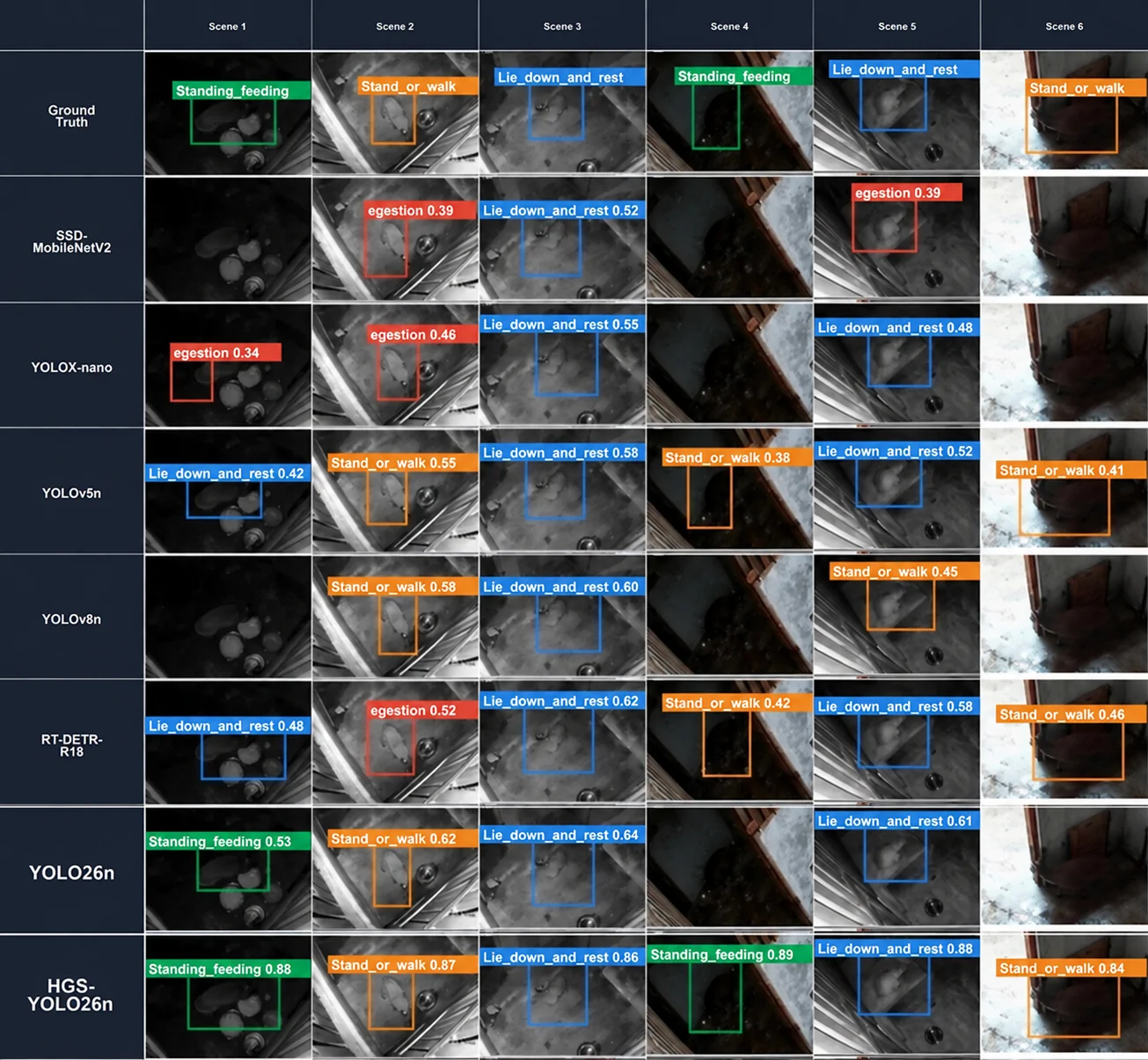
Detection examples from the compared models under weak illumination and occlusion.

**Figure 10:**
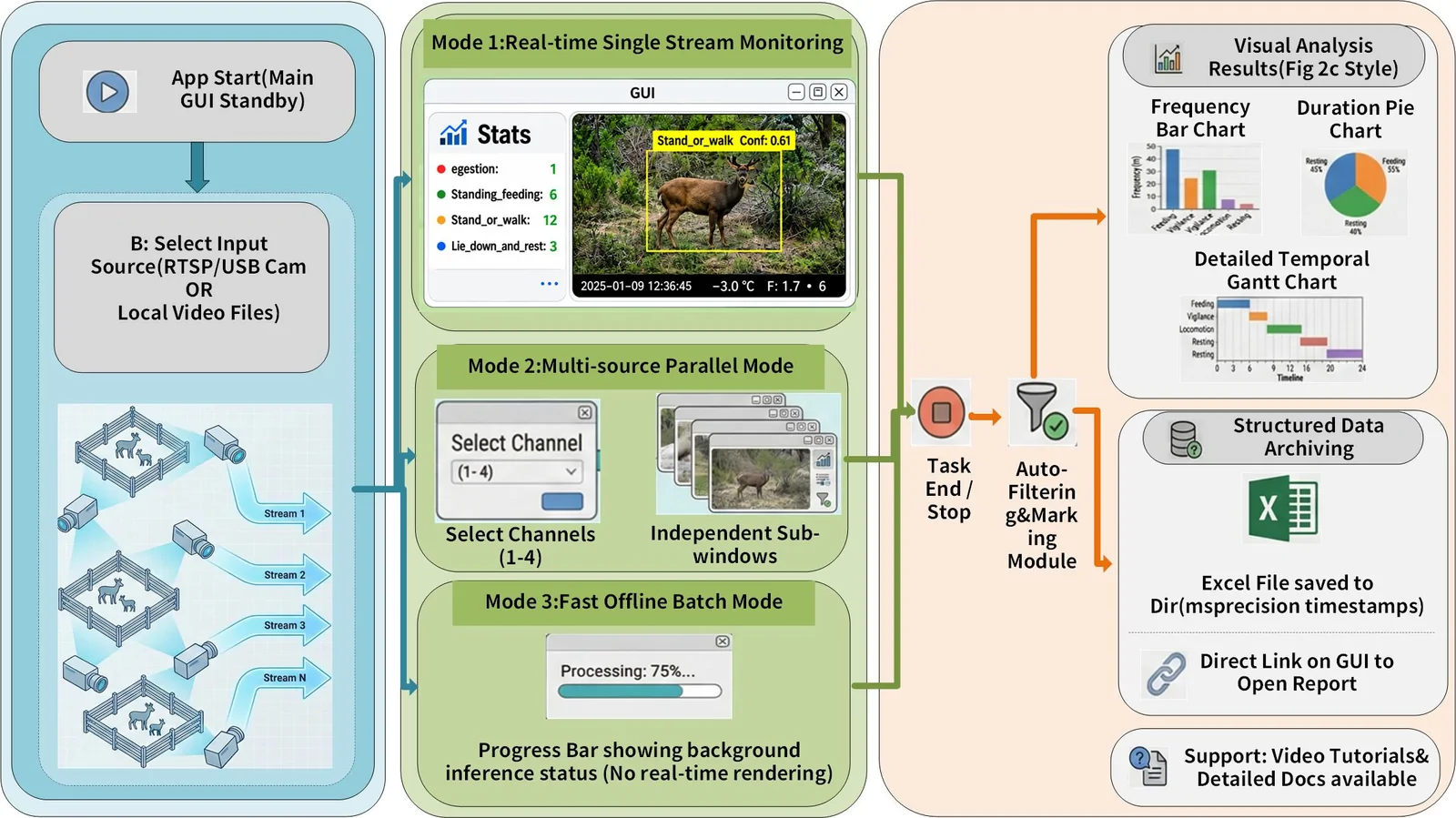
Workflow of the forest musk deer behavior detection and recording system.

**Figure 11:**
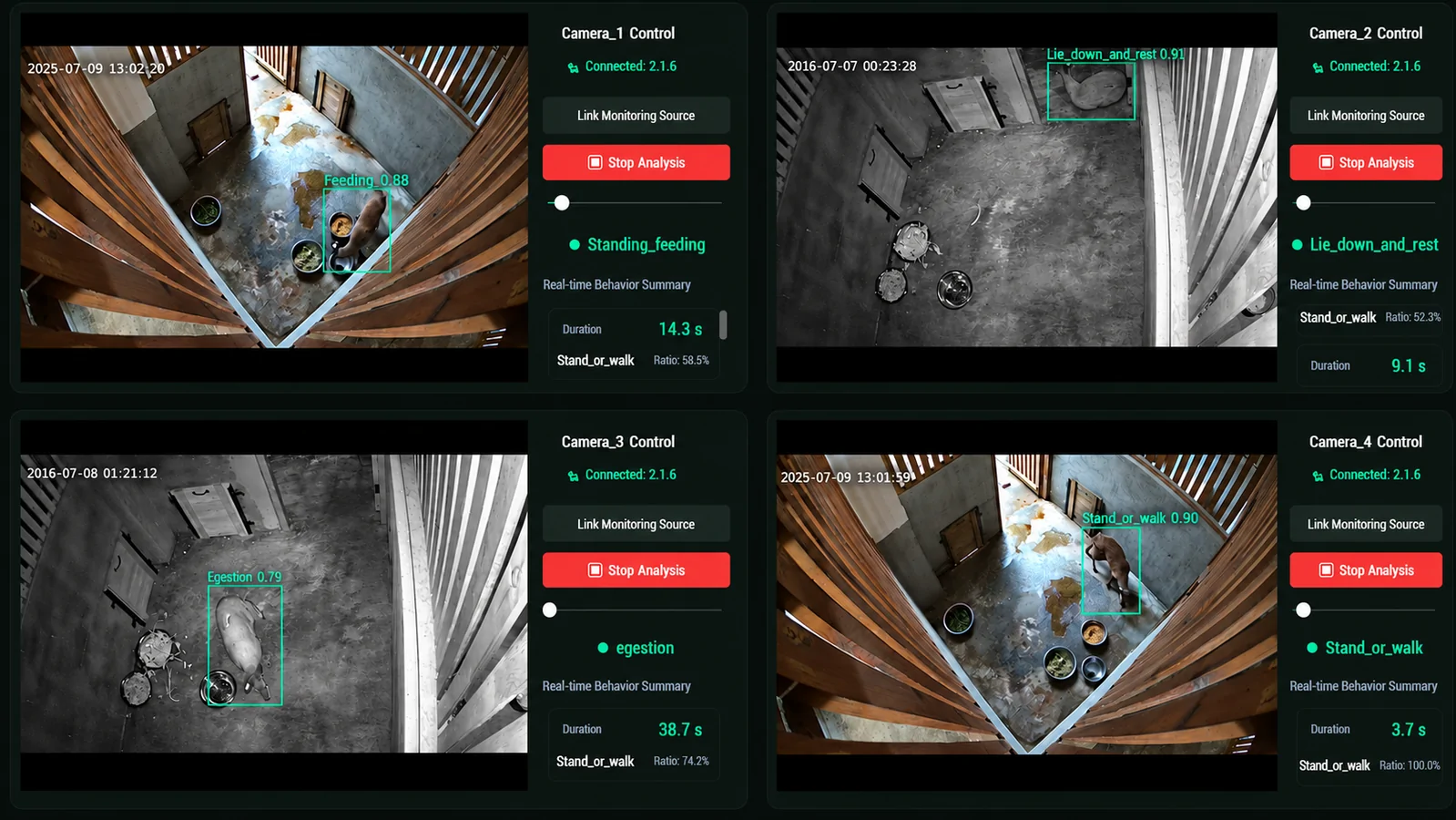
Multi-camera behavior monitoring interface.

**Figure 12:**
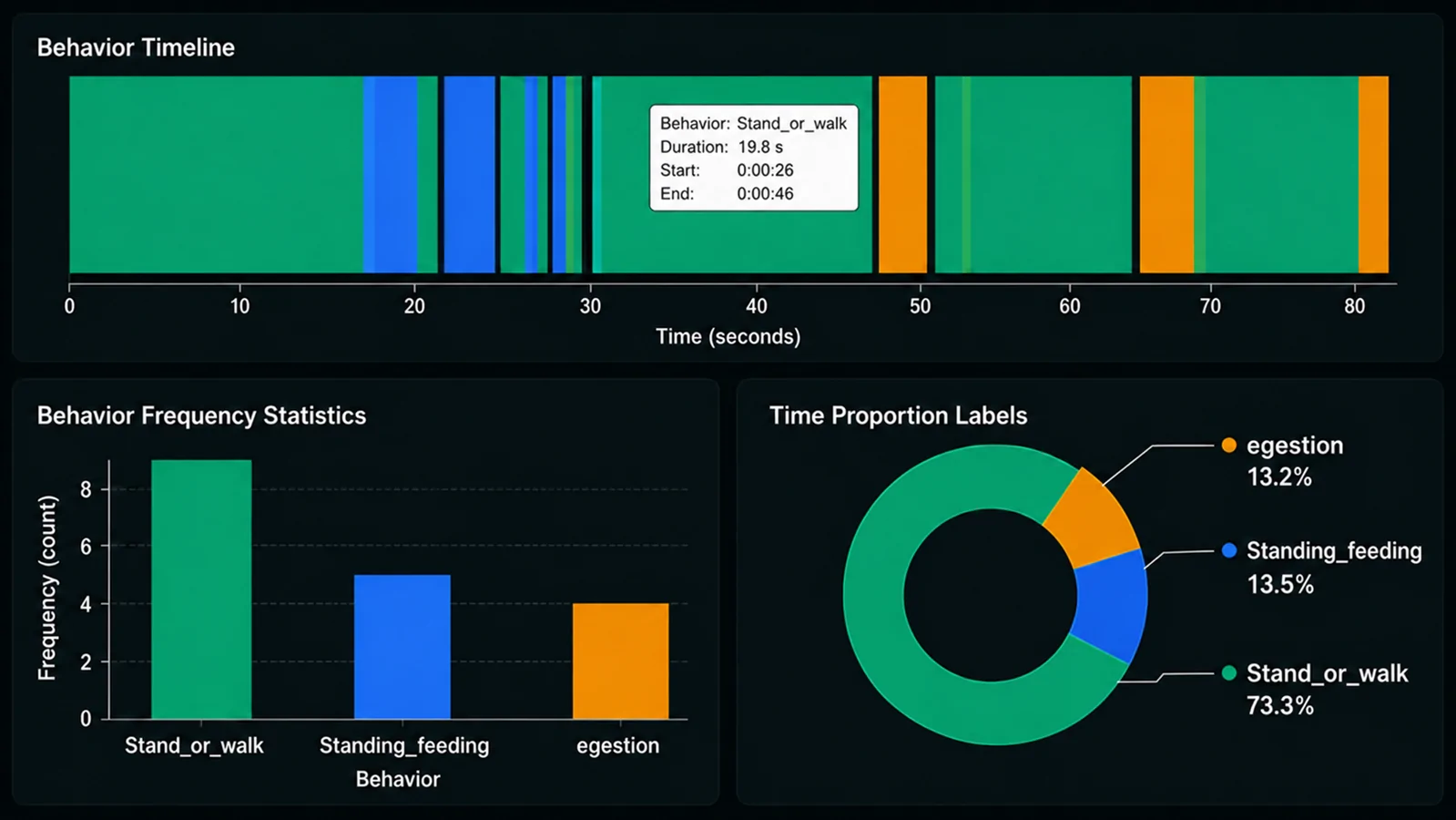
Behavior timelines, frequencies, and time proportions.

**Figure 13:**
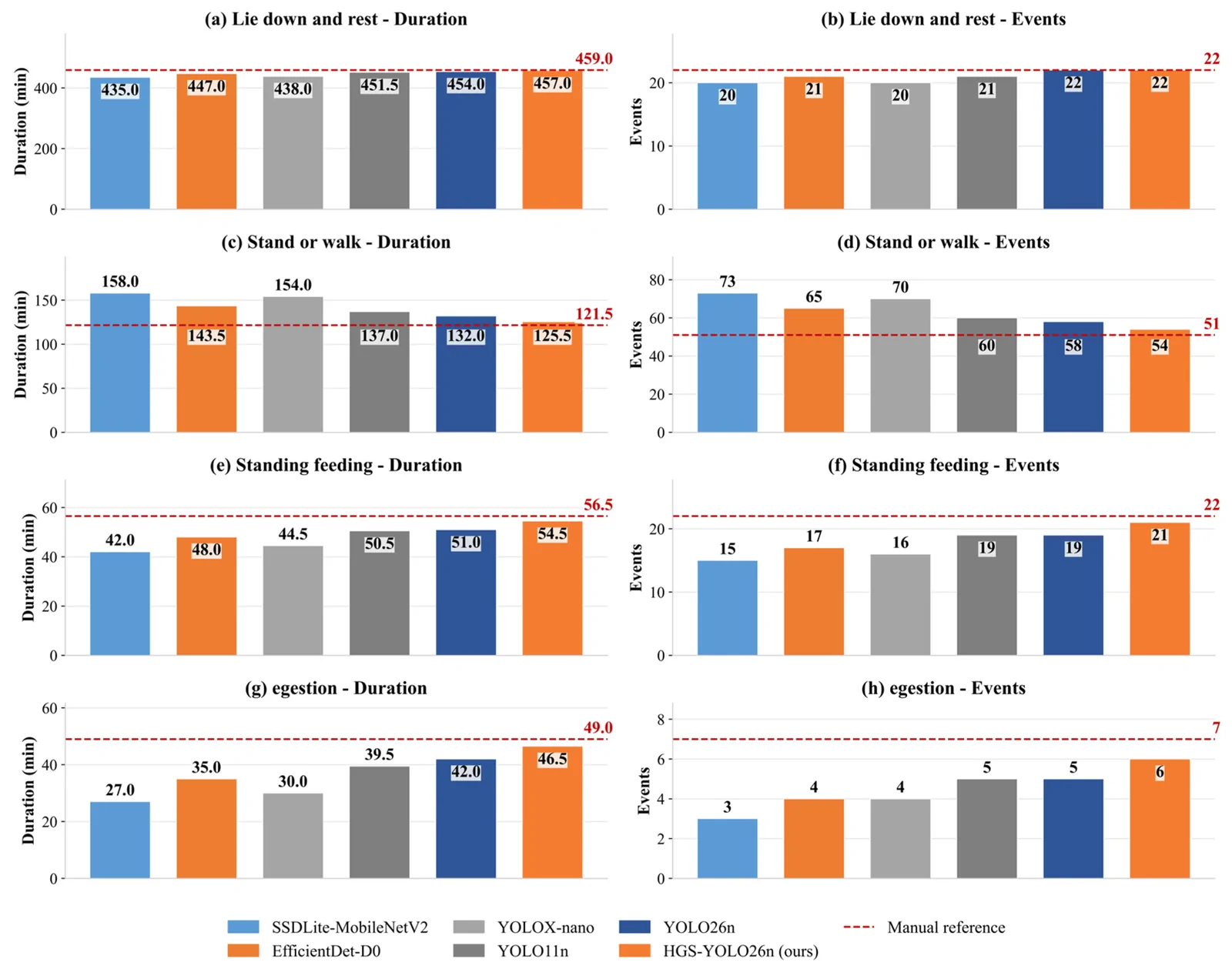
Behavior-duration and event-count estimates across five long videos.

**Table 1:** Operational criteria for behavior classification and annotation.

Category	Operational Definition	Handling of Borderline Samples
egestion	The hind limbs are flexed or the hindquarters are visibly lowered, with an identifiable excretion-related posture or visible excretory signs near the tail and hindquarters.	Ordinary standing, complete hindquarter occlusion, or unconfirmed excretion is not labeled as egestion. Samples are excluded when blur or occlusion prevents reliable assessment of the hind limbs and hindquarters.
Standing_feeding	The deer remains upright on four limbs with its head lowered toward the trough or feed. The muzzle contacts or is immediately adjacent to the feed, with a head–neck posture consistent with feeding.	Proximity to the feeding trough alone is insufficient. If feeding cues are unclear but an upright posture is confirmed, the sample is labeled as Stand_or_walk; otherwise, it is reviewed or excluded.
Stand_or_walk	The deer remains upright on four limbs without clear feeding- or egestion-related cues. This category includes stationary standing, slow walking, and brief upright transitional states.	Standing and walking are not separated because reliable discrimination requires displacement, speed, or trajectory information from consecutive frames.
Lie_down_and_rest	The body lies close to the ground and is supported by folded limbs, producing a low contour clearly distinct from an upright posture.	Brief head lowering, bending, or apparent body lowering caused by occlusion is not labeled as lying/resting. Samples are excluded when severe occlusion prevents reliable whole-body annotation.

*Note: The four categories were mutually exclusive. When class-specific feeding or egestion cues were absent, clearly upright animals were labeled as Stand_or_walk; indeterminate samples were excluded.*

**Table 2:** Dataset split and class distribution.

Dataset	Images	Labels	Egestion	Standing_Feeding	Stand_or_Walk	Lie_Down_and_Rest
Training	5412	5412	839	814	2276	1483
Validation	1348	1348	209	203	568	368
Total	6760	6760	1048	1017	2844	1851

**Table 3:** Main training and data-augmentation settings.

Item	Setting
Input size	640 × 640
Epochs	250
Batch size	8
Optimizer	AdamW
Initial learning rate	0.002
Weight decay	0.0005
Pretrained	False
AMP	False
Random seed	0
Mosaic probability	0.50
MixUp probability	0.08
Horizontal flip probability	0.50
Rotation	±4°
Translation	±8%
Scaling	0.65–1.35
Mosaic closing	final 12 epochs
HSV hue gain	0.015
HSV saturation gain	0.55
HSV value gain	0.40

**Table 4:** Detection results of HGS-YOLO26n for each behavior class.

Class	Precision	Recall	mAP50	mAP50-95
Overall	0.9652	0.9322	0.9721	0.8409
egestion	0.9870	0.9513	0.9812	0.8354
Standing_feeding	0.9044	0.9242	0.9523	0.8407
Stand_or_walk	0.9802	0.8742	0.9603	0.7945
Lie_down_and_rest	0.9894	0.9789	0.9945	0.8928

**Table 6:** Detection performance and computational efficiency of the compared real-time detectors.

Model	P	R	mAP50	mAP50-95	Params/M	GFLOPs	FPS
SSDLite-MobileNetV2	0.8843	0.8529	0.9024	0.7258	4.29	3.58	158.4
EfficientDet-D0	0.9124	0.8843	0.9320	0.7768	3.92	3.90	134.6
YOLOX-nano	0.8906	0.8671	0.9146	0.7415	0.91	2.55	226.8
YOLOX-tiny	0.9139	0.8875	0.9341	0.7692	5.06	15.20	176.2
PP-YOLOE-s	0.9392	0.9102	0.9585	0.8124	7.93	17.36	128.5
RTMDet-tiny	0.9361	0.9080	0.9540	0.8076	4.80	8.13	164.7
RT-DETR-R18	0.9458	0.9177	0.9623	0.8216	20.01	59.96	76.3
YOLOv5n	0.9193	0.8841	0.9365	0.7752	2.60	7.67	194.8
YOLOv8n	0.9257	0.8953	0.9441	0.7864	3.21	8.70	185.2
YOLO11n	0.9311	0.9015	0.9473	0.7923	2.59	6.53	178.6
YOLO26n	0.9432	0.9084	0.9528	0.8035	2.38	5.22	214.3
HGS-YOLO26n (ours)	0.9652	0.9322	0.9721	0.8409	2.84	5.86	176.8

**Table 7:** Video conditions and manually reviewed reference records for long-video application validation.

Video	Condition	Total Duration (min)	Effective/ Undetermined (min)	Lie_Down_ and_Rest (min/events)	Stand_or_ Walk (min/events)	Standing_ Feeding (min/events)	Egestion (min/events)
V1	Ordinary surveillance	135.0	124.0/11.0	74.0/4	24.0/10	17.0/5	9.0/1
V2	Ordinary surveillance	160.0	145.5/14.5	101.0/5	24.5/9	9.0/4	11.0/1
V3	Ordinary surveillance	150.0	140.0/10.0	88.0/4	29.0/12	10.5/5	12.5/2
V4	Ordinary surveillance	130.0	120.0/10.0	72.0/4	27.0/9	14.0/5	7.0/1
V5	Nighttime weak light	180.0	156.5/23.5	124.0/5	17.0/11	6.0/3	9.5/2
Total	—	755.0	686.0/69.0	459.0/22	121.5/51	56.5/22	49.0/7

## Data Availability

The datasets generated and/or analyzed during the current study are available from the corresponding author upon reasonable request. The raw surveillance videos cannot be publicly released due to the protection requirements associated with a national first-class protected wildlife species, as well as confidentiality considerations related to the breeding facility and continuous monitoring environment. Processed data, including selected images and annotations used for model development and evaluation, may be made available for academic research purposes upon reasonable request and with approval from the data owner. Researchers interested in developing automated behavior monitoring methods for captive forest musk deer are also welcome to participate in collaborative evaluations, and submitted models may be tested on available evaluation data subject to relevant agreements.
